# Genetic Engineering of Umbilical Cord-Derived Mesenchymal Stem Cells to Enhance BMP-2 Secretion via Signal Peptide Optimization

**DOI:** 10.3390/biomedicines14010076

**Published:** 2025-12-30

**Authors:** Nuzli Fahdia Mazfufah, Ismail Hadisoebroto Dilogo, Retno Wahyu Nurhayati, Delvac Oceandy, Silvia Tri Widyaningtyas, Maulana Dias Pratama, Goo Jang

**Affiliations:** 1 Doctoral Program in Biomedical Sciences, Faculty of Medicine, Universitas Indonesia, Jakarta 10430, Indonesia; nuzlifahdia@gmail.com; 2 Stem Cell and Tissue Engineering Research Center, Indonesia Medical Education and Research Institute, Faculty of Medicine, Universitas Indonesia, Jakarta 10430, Indonesia; ismailortho@gmail.com (I.H.D.); delvac.oceandy@manchester.ac.uk (D.O.); maulana.dias.pratama99@gmail.com (M.D.P.); 3 Department of Orthopedic and Traumatology, Faculty of Medicine, Universitas Indonesia–Dr. Cipto Mangunkusumo National General Hospital, Jakarta 10430, Indonesia; 4 Stem Cell Medical Technology Integrated Service Unit, Dr. Cipto Mangunkusumo National General Hospital, Jakarta 10430, Indonesia; 5 Department of Chemical Engineering, Faculty of Engineering, Universitas Indonesia, Depok 16424, Indonesia; 6 Division of Cardiovascular Science, Manchester Academic Health Science Center, University of Manchester, Manchester M13 9PG, UK; 7 Virology and Cancer Pathobiology Research Center, Faculty of Medicine, Universitas Indonesia, Jakarta 10430, Indonesia; silvia.tw@ui.ac.id; 8 Department of Veterinary Medicine, College of Veterinary Medicine, Seoul National University, Seoul 08826, Republic of Korea; snujang@snu.ac.kr

**Keywords:** mesenchymal stem cells, bone morphogenetic protein 2, secretome, signal peptide, transfection

## Abstract

**Background/Objectives**: Mesenchymal stem cells (MSCs) are recognized for their therapeutic potential due to their ability to secrete bioactive molecules. Among these secreted factors, bone morphogenetic protein-2 (BMP-2) is known as a secreted factor that plays a crucial role in bone healing and regeneration. However, MSCs naturally secrete only small amounts of BMP-2. To improve the bone healing capacity of MSCs, it is essential to enhance the secretion of BMP-2 in MSCs. One approach that can be used to achieve this goal is by genetically engineering MSCs. Incorporating signal peptides (SPs) into the inserted gene sequence can significantly improve protein secretion efficiency. In this proof-of-concept study, we explored the role of SPs in optimizing BMP-2 secretion in umbilical cord-derived MSCs; **Methods**: Three human-derived SPs, namely glial-derived neurotrophic factor (GDNF), chemotactic antibacterial glycoprotein 7 (CAP7), and platelet-derived growth factor subunit B (PDGFB), were selected. Transfection of MSCs was performed using polyethylenimine, Lipofectamine 2000^®^, and Lipofectamine 3000^®^. Transfection efficiency confirmed based on Green Fluorescence Protein expression. BMP-2 secretion levels were quantified using an ELISA assay; **Results**: Lipofectamine 3000^®^ achieved the highest transfection efficiency, reaching approximately 10%. BMP-2 secretion levels varied significantly depending on the SPs used, with PDGFB yielding the highest BMP-2 concentration (279.21 ± 6.91 pg/mL), followed by GDNF (265.65 ± 11.49 pg/mL) and CAP7 (233.72 ± 32.33 pg/mL); **Conclusions**: These findings demonstrate that SP selection critically influences BMP-2 secretion efficiency in genetically engineered MSCs and underscore its potential to enhance the therapeutic applicability of MSC-based strategies for bone healing.

## 1. Introduction

Mesenchymal stem cells (MSCs) have been investigated for many years for their potential for regenerative therapy. Initial therapy of MSCs focused on the transplantation of viable cells [1]; however, in recent years, accumulating evidence indicates that their regenerative effects are largely mediated by their secreted factors, known as secretome [2,3]. The MSC secretome consists of growth factors, cytokines, extracellular matrix proteins, exosomes, microRNAs, and other signalling molecules [4,5]. Among these secreted factors, bone morphogenetic protein-2 (BMP-2) is known as a secreted factor that plays a crucial role in bone healing and regeneration [4]. Recombinant human BMP-2 (rhBMP-2) was authorized by the U.S. Food and Drug Administration (FDA) in 2002 for use in certain fracture healing and spinal fusion surgeries [5]. Since then, rhBMP-2 has been widely used as a treatment for bone-related problems [5].

In the standard culture condition, MSCs only secrete a limited quantity of BMP-2, which limits their therapeutic efficacy in bone healing [6,7]. Therefore, current bone healing therapeutic strategies primarily use MSC secretome in combination with exogenous rhBMP-2 [8]. The clinical use of exogenous rhBMP-2 has several limitations, including dose-related adverse effects such as inflammation and ectopic bone formation [9]. In addition, exogenous rhBMP-2 is typically produced in prokaryotic expression systems, such as *Escherichia coli*, which poses various challenges, including the high cost of purification and the lack of post-translational modification mechanisms that often lead to misfolded, unstable, or non-functional eukaryotic proteins [10].

To improve the bone healing capacity of MSCs and diminish the dependence on exogenous rhBMP-2, it is essential to enhance the secretion of BMP-2 in MSCs. One approach that can be used to achieve this goal is by genetically engineering MSCs by inserting the *BMP-2* gene into the cells. Several studies have shown that incorporating a signal peptide (SP) into the inserted gene sequence can significantly enhance protein secretion [11,12,13,14,15]. SPs are short amino acid sequences located at the N-terminus of proteins that act as “address tags,” directing newly synthesized proteins into the secretory pathway [16]. The selection of an appropriate SP is important because it affects the efficiency of protein folding, processing, and transport [17,18,19,20]. Without an appropriate SP, the protein may be retained within the cell or misdirected to incorrect compartments [16].

However, there has been no systematic evaluation of optimized SP to enhance BMP-2 secretion specifically in MSCs, and the effect of SP selection on BMP-2 secretion in the MSC secretome remains poorly defined. Therefore, this study aimed to address this gap by incorporating selected optimized SP into the *BMP-2* sequence and expressing the constructs in MSCs to evaluate BMP-2 secretion. The findings from this work may inform future strategies for enhancing the regenerative potential of MSC-based therapies, particularly in applications where targeted protein secretion is critical for therapeutic success.

## 2. Methods

### 2.1. Study Design

This study was an in vitro experimental investigation. Based on a preliminary bioinformatic study, 3 SPs found in secreted proteins were selected: (i) glial-derived neurotrophic factor human (GDNF_HUMAN), (ii) chemotactic antibacterial glycoprotein 7 (CAP7_HUMAN), and (iii) platelet-derived growth factor subunit B (PDGFB_HUMAN). The native SP of BMP-2 (BMP_HUMAN) was used as the control.

**Figure 1:**
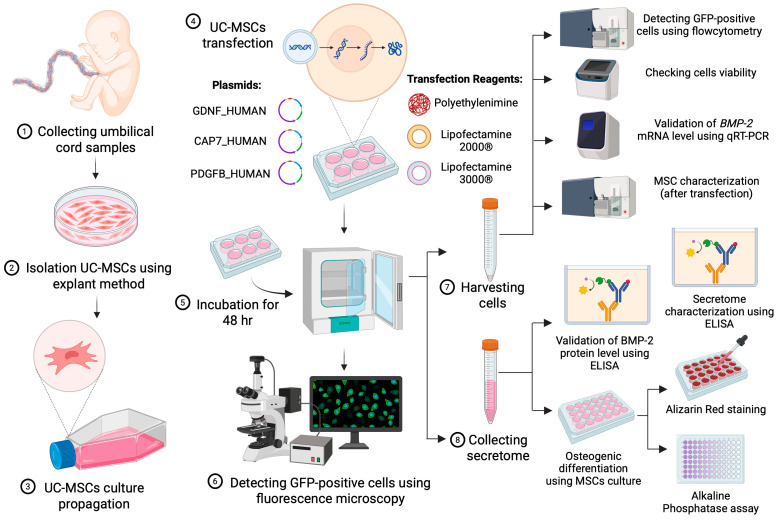
Overview of the experimental workflow. The workflow illustrates key steps, including UC-MSCs isolation, plasmid construction, gene transfection and evaluation, secretome production, BMP-2 mRNA and protein quantifications, and secretome-BMP-2 functional assay in osteogenic differentiation of untransfected cells. (Created in BioRender. Fahdia, N. (2026). https://BioRender.com/2bdayt7).

The plasmids were constructed by fusing SPs amino acid sequence in BMP-2 protein. The plasmids were transfected into umbilical cord-derived MSCs using either lipid-based or polymer-based reagents. Positive transfected cells were confirmed based on Green Fluorescent Protein (GFP) expression, which was qualitatively visualized under a fluorescence microscope and quantitatively analyzed with a flow cytometer. The expression of BMP-2 post-transfection was evaluated at the relative mRNA level using qRT–PCR and at the protein level using an enzyme-linked immunosorbent assay (ELISA). The MSC phenotype was characterized pre- and post-transfection using flow cytometry, while the secretome profile after transfection was analyzed using ELISA to quantify the concentrations of vascular endothelial growth factor (VEGF), transforming growth factor-beta 1 (TGF-β1), and interleukin-10 (IL-10). Finally, osteogenic differentiation assays were performed to assess the biological function of the recombinant BMP-2 protein secreted by engineered cells in untransfected MSCs, as confirmed by alizarin red staining and alkaline phosphatase (ALP) activity assay. The overall experimental workflow is illustrated in Figure 1.

### 2.2. Isolation and Culture of MSCs

All procedures in this study have been reviewed and approved by the Health Research Ethics Committee of Faculty of Medicine, Universitas Indonesia. UC-MSCs were isolated from umbilical cords obtained from cesarean deliveries using the explant method, as previously described [21]. Umbilical cord samples were collected from three healthy donors after informed consent, with inclusion criteria defined as full-term deliveries (≥37 weeks of gestation), absence of maternal infectious diseases or pregnancy-related complications, and no history of chronic inflammatory or autoimmune conditions. Each experiment in this study was conducted in cells isolated from 3 different donors, with each trial performed in triplicate.

UC-MSCs were cultured in Alpha Minimum Essential Medium (αMEM) supplemented with penicillin–streptomycin, amphotericin B, GlutaMAX™ Supplement, and 10% platelet-rich plasma (Indonesian Red Cross Society, Jakarta, Indonesia). Cultures were maintained at 37 °C in a humidified atmosphere with 5% CO_2_. The culture medium was replaced every 2–3 days until the cells reached approximately 80–90% confluence. Cell viability was determined using trypan blue exclusion and quantified with a hemocytometer. All reagents and media components were purchased from Thermo Scientific (Philadelphia, PA, USA).

### 2.3. Plasmid Transfections

**Figure 2:**
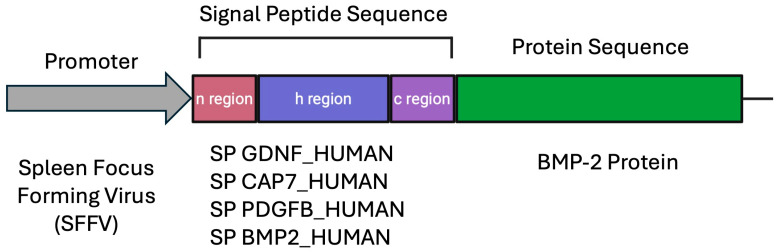
Design of the plasmid incorporating a signal peptide in the BMP-2 sequence. The SP sequence is located at the N-terminus of the BMP-2 amino acid sequence and consists of the N-, H-, and C-regions. The SP sequence is directly fused to the BMP-2 sequence to facilitate efficient targeting into the secretory pathway.

Plasmid constructs were designed using the VectorBuilder platform (https://en.vectorbuilder.com, accessed on 6 February 2024) (Figure 2). Each plasmid consisted of (i) spleen focus-forming virus (SFFV) promoter driving the expression of the gene of interest, (ii) open reading frame (ORF) containing the amino acid sequence of the optimized SP fused to the BMP-2 protein sequence, and (iii) GFP marker for visualization of transfection efficiency. SP sequences were obtained from the Signal Peptide database (http://www.signalpeptide.de accessed on 6 February 2024) according to Mazfufah et al. [22] and are listed in Table 1.

All plasmids were propagated in *Escherichia coli* with ampicillin as a screening antibiotic. Positive transformant from a single colony was inoculated into LB broth containing 1 mg/mL ampicillin, followed by incubation at 37 °C under continuous shaking (16 h). Plasmid DNA was extracted using the Presto™ Mini Plasmid Kit (Geneaid, New Taipei City, Taiwan) according to the manufacturer’s instructions. The purity and concentration of the isolated DNA were determined using Varioskan™ Microplate Reader (Thermo Scientific, USA) at 260/280 nm. The purified plasmid DNA was subsequently transfected into UC-MSCs.

UC-MSCs at passage 2 were seeded in 12-well plates at a density of 5000 cells/cm^2^. Previous experiments indicated that MSCs with high passage numbers exhibited reduced transfection efficiency [23]. When the cultures reached 60–70% confluence, the cells were prepared for transfection and maintained in serum/antibiotic-free αMEM. Plasmid transfection was carried out using 3 different transfection reagents: polyethylenimine (PEI), Lipofectamine 2000^®^, and Lipofectamine 3000^®^, according to the manufacturer’s protocols. Plasmid DNA was diluted with OPTI-MEM and the P3000™ reagent (specific for Lipofectamine 3000^®^). In a separate tube, each transfection reagent was diluted in OPTI-MEM. The 2 solutions were then combined at a 1:1 ratio and incubated at room temperature for 15 min to allow the formation of DNA–lipid complexes. The transfection mixture was then added to the cultured cells. Details of DNA amounts and transfection reagent volumes are provided in Supplementary Materials. Transfected cells were cultured under standard incubation conditions for 48 h and subsequently used for further analyses.

### 2.4. Analysis of the GFP Positive Signal

Transfection efficiency was evaluated based on the expression of the GFP reporter. Cells exhibiting GFP fluorescence were observed under a fluorescence microscope, where successful transfection was indicated by distinct green fluorescence within the cytoplasm of the transfected cells.

#### 2.4.1. GFP Qualitative Analysis Using Fluorescence Microscopy

Transfected cells were observed directly on the culture flasks, without cell detachment, using a Nikon Eclipse Ni-U fluorescence microscope (Nikon, Tokyo, Japan). Prior to imaging, the culture medium was removed, and the cells were gently rinsed twice with phosphate-buffered saline (PBS, pH 7.4) to eliminate debris and residual transfection reagents. Images were captured using identical exposure settings to ensure consistency across samples.

#### 2.4.2. GFP Quantitative Analysis Using Flow Cytometry

Cells were detached with trypsin and then washed twice with PBS (pH 7.4). Flow cytometry analysis was performed using a BDFACS ARIAIII™ (BD Bioscience, San Jose, CA, USA) equipped with a 488 nm excitation laser and a 530/30 nm emission filter for GFP detection. A minimum of 10,000 events was acquired per sample. Non-transfected UC-MSCs were used as negative controls to set the gating parameters. The percentage of GFP-positive cells was used to determine transfection efficiency in the cell population.

### 2.5. BMP-2 Gene Expression

Total RNA was extracted from UC-MSCs using the Quick-RNA Miniprep Kit (Zymo Research, Irvine, CA, USA) according to the manufacturer’s instructions. The purity and concentration of RNAs were measured based on absorbance at 260/280 nm. Samples with an acceptable ratio of 1.8 to 2.0 were selected for the next reverse transcription reaction. Samples were synthesized into cDNA using ReverTra Ace qPCR RT Master Mix with gDNA Remover (Toyobo, Osaka, Japan) according to the manufacturer’s instructions. qRT-PCR was performed using SensiFAST™ SYBR^®^ Lo-ROX (Bioline, London, UK) on an Applied Biosystems^®^ 7500 Fast (Thermo Scientific, USA). Primer sequences for *BMP-2* (target gene) and *GAPDH* (housekeeping gene) are listed in Table 2. Melting curve analysis was conducted to verify the specificity of the amplified products, and relative gene expression levels were calculated using the 2^−ΔΔCt^ (Livak method).

### 2.6. Isolation and BMP-2 Protein Quantification of Secretome from Engineered MSCs

Transfected cells were cultured in serum/antibiotic-free αMEM for 48 h post-transfection. The conditioned medium was centrifuged at 3500 rpm for 30 min at 4 °C to remove cell debris and suspended particles. The resulting supernatant was carefully transferred to a new sterile tube and filtered through a 0.22-μm membrane filter to ensure sterility and clarity of the secretome sample. Quantification of BMP-2 protein in the secretome was performed using a human BMP-2 ELISA kit (Elabscience, Wuhan, China) according to the manufacturer’s protocol. Briefly, 100 µL of each sample and standard were added to the precoated wells and incubated at 37 °C, followed by washing and sequential addition of biotinylated detection antibody, HRP-conjugated streptavidin, and substrate solution. The reaction was terminated with the stop solution, and absorbance was measured at 450 nm. BMP-2 concentrations were calculated from a standard curve.

### 2.7. Characterization Post-Transfection

#### 2.7.1. Phenotypic Profile of UC-MSCs Post-Transfection

Characterization of UC-MSCs pre- and post-transfection was performed using flow cytometry to evaluate their immunophenotypic profile. At 48 h after transfection, cells were harvested and washed with PBS. Surface marker expression was assessed using the BD Stemflow™ Human MSC Analysis Kit (BD Biosciences, San Diego, CA, USA), which includes antibodies against CD73 (APC), CD90 (FITC), and CD105 (PerCP-Cy-5) as positive markers, and CD34, CD45, and HLA-DR as negative markers (PE). Cells were incubated with the antibody cocktail for 30 min at 4 °C in the dark, followed by washing with PBS to remove excess antibodies. The population was selected using FSC-A versus SSC-A to exclude aggregates and dead cells. Corresponding isotype controls were used to determine background fluorescence.

#### 2.7.2. Secretome Profile of UC-MSC Post-Transfection

Secretome profiling after transfection was performed using ELISA to quantify the levels of key cytokines and growth factors, including VEGF, TGF-β1, and IL-10. ELISA was performed using a human VEGF kit (Elabscience, China), a human TGF-β1 kit (Elabscience, China), and a human IL-10 kit (Elabscience, China) according to the manufacturer’s protocol.

### 2.8. Osteogenic Differentiation

Osteogenic differentiation was evaluated to assess the ability of the BMP-2 secretome to induce osteogenesis in vitro using UC-MSCs. Based on preliminary findings, UC-MSCs at passages 2–7 exhibited consistent osteogenic potential; therefore, passage 5 cells were used in this experiment. UC-MSCs at passage 5 were seeded in 24-well plates at a density of 5000 cells/cm^2^ and cultured until approximately 70% confluence. The growth medium of UC-MSCs was then replaced with the StemPro™ Osteogenesis Differentiation Kit (Thermo Scientific, USA). The BMP-2 secretome was added to the cells at a 1:1 ratio with osteogenesis medium, representing a fixed-volume application. For positive control, recombinant human BMP-2 Novosis^®^ (Daewoong Pharmaceutical, Seoul, Republic of Korea) was used at a concentration of 280 pg/mL (the selection concentration was determined based on the results showing the highest ELISA-quantified BMP-2 levels in the secretome of engineered MSCs). Differentiation cultures were maintained at 37 °C in a humidified incubator with 5% CO_2_ for 21 days, with the medium replaced every 2–3 days to support continuous differentiation.

### 2.9. Alizarin Red Staining

After 21 days of osteogenic differentiation, calcium phosphate deposition was visualized using 1% Alizarin Red S staining (pH 4.2) (Sigma-Aldrich, Saint Louis, MO, USA). The red-stained calcium deposits were examined under an inverted microscope (Nikon, Japan) to confirm mineralization. For quantitative analysis, the bound dye was extracted using a solution of ddH_2_O containing 20% methanol and 10% acetic acid, followed by incubation at room temperature for 15 min. The resulting suspension was measured at 562 nm to quantify the extent of calcium mineralization.

### 2.10. ALP Activity

ALP activity was determined using the ALP Activity Assay Kit (Abnova, Taipei, Taiwan) following the manufacturer’s protocol. The working solution was freshly prepared by mixing the assay buffer with 5 mM magnesium acetate and 10 mM p-nitrophenyl phosphate liquid substrate. Each 50 µL of sample was added to a 96-well plate, followed by the addition of 150 µL of the working solution. The absorbance or optical density was measured at 405 nm at 0 and 4 min after the addition of the working solution. The change in absorbance over time was used to calculate ALP enzymatic activity, which was expressed as International Units per Liter (IU/L).

### 2.11. Statistical Analysis

Statistical analysis was performed using GraphPad Prism 10 (GraphPad Software, Boston, MA, USA). Data normality was assessed using the Shapiro–Wilk test (*n* < 30; *p* > 0.05 indicating normal distribution), and homogeneity of variance was evaluated using Levene’s test (*p* > 0.05 indicating homogeneity). Data satisfying both assumptions were analyzed using one-way ANOVA, followed by Tukey’s post hoc test to determine intergroup differences. For data that did not meet the assumptions of normality or homogeneity, even after transformation, the nonparametric Kruskal–Wallis test was applied, followed by the Mann–Whitney U test for pairwise comparisons. Results are presented as mean ± standard deviation (SD), and statistical significance was set at *p* < 0.05. All data were collected from triplicate experiments, and every experiment was tested using cells collected from 3 unrelated donors.

## 3. Results

### 3.1. Optimization of UC-MSC Transfection Efficiency Using Different Transfection Reagents

**Figure 3:**
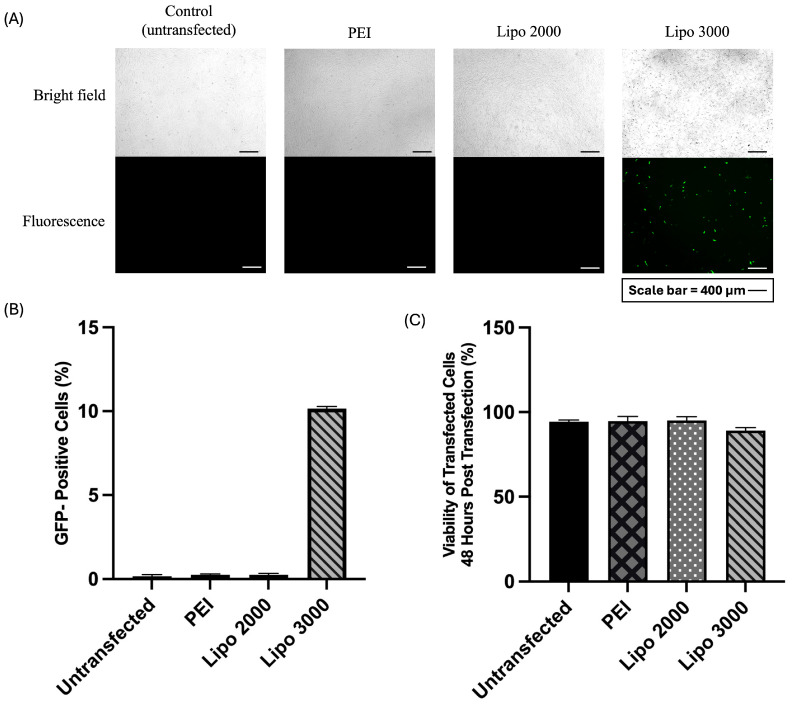
Optimization of UC-MSCs transfection using different transfection reagents: (**A**) Representative brightfield and fluorescence microscopy images showing GFP expression in UC-MSCs transfected with different reagents compared with untransfected control cells. (**B**) Quantitative analysis of GFP expression (%) showing significantly higher transfection efficiency in the Lipofectamine 3000^®^ group compared to other reagents. (**C**) Cell viability (%) after transfection, demonstrating that all reagents maintained over 80% cell viability. Data are presented as mean ± SD.

Different DNA concentration-to-transfection reagent volume ratios for the three transfection reagents are provided in the Supplementary Material. Among 3-tested transfection reagents, only Lipofectamine 3000^®^ successfully expressed GFP in UC-MSCs, with approximately 10% GFP-positive cells at 48 h post-transfection (Figure 3A,B). In contrast, PEI and Lipofectamine 2000^®^ demonstrated negligible transfection efficiency (<1%). Lipofectamine 3000^®^ also maintained a high cell viability of >90% (Figure 3C), suggesting minimal cytotoxic effects. These results indicate that Lipofectamine 3000^®^ is the most efficient and least toxic reagent for gene delivery in UC-MSCs compared with PEI and Lipofectamine 2000^®^.

### 3.2. Evaluation of Transfection Efficiency, Cell Viability, and BMP-2 Secretion in UC-MSCs Transfected with Different Signal Peptide Constructs

**Figure 4:**
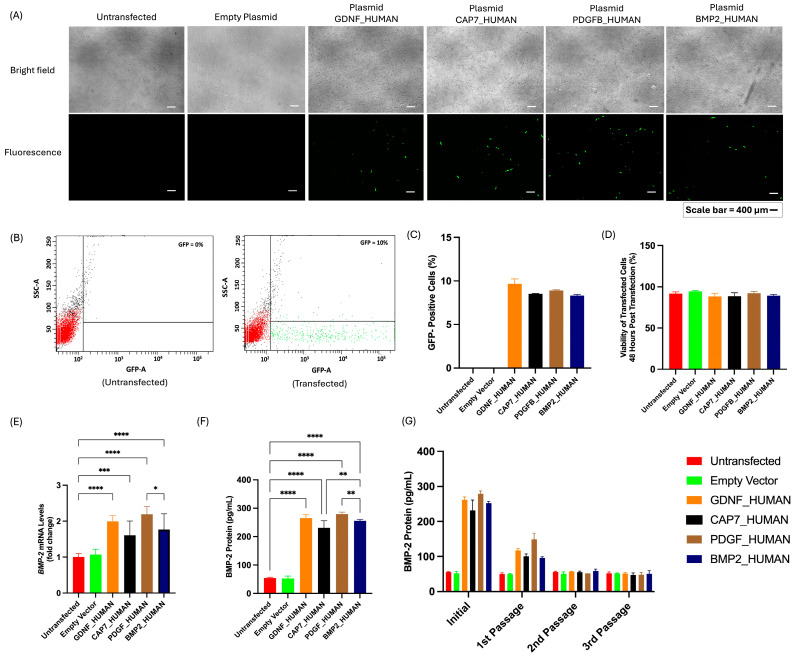
Evaluation of transfection efficiency, cell viability, and BMP-2 secretion in UC-MSCs transfected with different signal peptide constructs: (**A**) Representative brightfield and fluorescence microscopy images showing GFP expression. (**B**) Representative flow cytometry dot plots showing GFP-negative (untransfected) and GFP-positive (transfected) UC-MSC populations. (**C**) Quantitative analysis of GFP expression (%). (**D**) Cell viability (%) after transfection. (**E**) Relative *BMP2* mRNA expression quantified by qRT-PCR and (**F**) BMP-2 protein secretion quantified by ELISA at 48 h post-transfection. (**G**) BMP-2 protein levels in UC-MSC secretome across serial passages. Data are presented as mean ± SD. Statistical significance was determined using one-way ANOVA followed by Tukey’s post hoc test; *p* < 0.05 (*), *p* < 0.01 (**), *p* < 0.001 (***), *p* < 0.0001 (****).

Fluorescence microscopy images showed GFP expression at 48 h post-transfection (Figure 4A). GFP-positive signals were observed in all SP-transfected groups, whereas no GFP signal was detected in the untransfected and empty vector control groups. Flow cytometry analysis confirmed an average transfection efficiency of approximately 10% in all SP-transfected groups (Figure 4B,C). Cell viability remained above 90% across all groups, indicating that the transfection procedure did not induce significant cytotoxicity (Figure 4D).

A qRT-PCR analysis demonstrated that relative BMP-2 mRNA expression was significantly higher in UC-MSCs transfected with PDGFB_HUMAN construct (2.19 ± 0.22 folds) compared to other constructs: GDNF_HUMAN (1.99 ± 0.16 folds), CAP7_HUMAN (1.61 ± 0.40 folds), and BMP2_HUMAN (1.77 ± 0.44 folds) (Figure 4E). Consistent with the mRNA results, ELISA quantification showed promoted BMP-2 protein secretion in PDGFB_HUMAN group, reaching 279.21 ± 6.91 pg/mL, which was significantly higher than GDNF_HUMAN (265.65 ± 11.49 pg/mL), CAP7_HUMAN (233.72 ± 32.33 pg/mL), and BMP2_HUMAN (255.36 ± 5.22 pg/mL) (Figure 4F). However, serial passage analysis demonstrated a gradual decline in BMP-2 secretion after cell subculture in all SP-transfected groups, suggesting a transient expression pattern typical of plasmid-based transfection (Figure 4G).

### 3.3. Phenotypic Profile and Secretome Profile of UC-MSCs Post-Transfection

**Figure 5:**
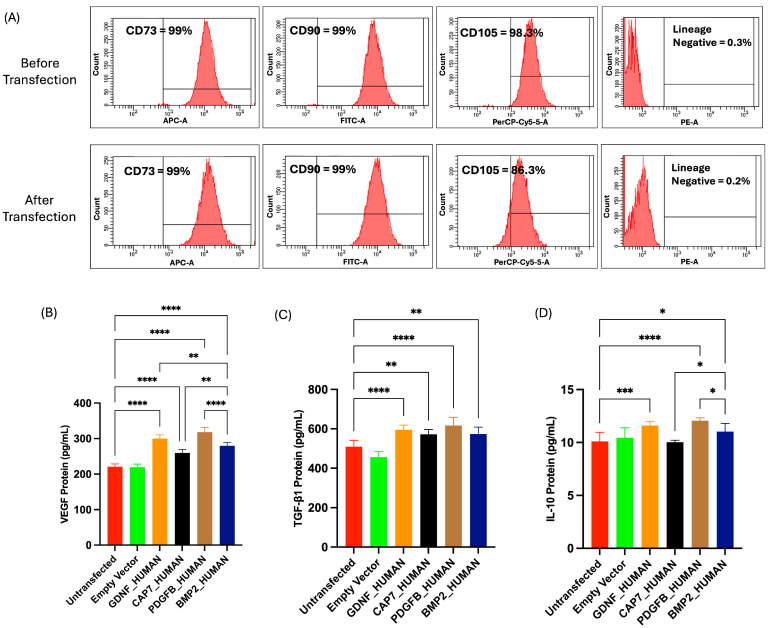
Phenotypic profile and secretome profile of UC-MSCs post-transfection: (**A**) Flow cytometry analysis of UC-MSCs’ surface marker expression (%) before and after transfection. (**B**–**D**) Quantitative ELISA analysis of (**B**) VEGF, (**C**) TGF-β1, and (**D**) IL-10 concentrations in UC-MSC secretome collected 48 h post-transfection. Data are presented as mean ± SD. Statistical significance was determined using one-way ANOVA followed by Tukey’s post hoc test; *p* < 0.05 (*), *p* < 0.01 (**), *p* < 0.001 (***), *p* < 0.0001 (****).

Flow cytometry analysis confirmed that UC-MSCs pre-transfection consistently expressed high levels of MSC-specific markers CD73, CD90, and CD105, while maintaining low expression of lineage-negative markers (<1%), confirming the MSC identity and purity of the cells. However, the data demonstrated a slight decrease in surface marker expression following transfection, particularly for CD105. This reduction was observed across all samples, with CD105 expression declining from >95% pre-transfection to <95% post-transfection. In contrast, CD73 and CD90 expression levels remained relatively stable, ranging between 95–100% before and after transfection (Figure 5A).

Characterization of the secretome profile following transfection was performed by measuring the concentrations of VEGF, TGF-β1, and IL-10 (Figure 5B–D). VEGF secretion (Figure 5B) was significantly increased in all groups transfected with SP plasmids compared to both the untransfected and empty vector controls (*p* < 0.05). The highest VEGF concentration was observed in cells transfected with PDGFB_HUMAN construct (318.19 ± 13.56 pg/mL) and GDNF_HUMAN construct (300.12 ± 10.69 pg/mL), which showed markedly elevated levels relative to the untransfected group (220.86 ± 8.01 pg/mL). Similarly, TGF-β1 secretion (Figure 5C) was enhanced following transfection with PDGFB_HUMAN (617.00 ± 41.47 pg/mL), GDNF_HUMAN (594.78 ± 24.49 pg/mL), BMP2_HUMAN (573.67 ± 34.92 pg/mL), and CAP7_HUMAN (594.78 ± 24.49 pg/mL) constructs, all of which showed significantly higher levels compared to the untransfected controls (*p* < 0.01 to *p* < 0.0001). Furthermore, IL-10 secretion (Figure 5D) also increased modestly but significantly after gene transfection (*p* < 0.05 to *p* < 0.0001). Among the groups, PDGFB_HUMAN-transfected cells exhibited the highest IL-10 level (12.06 ± 0.27 pg/mL).

### 3.4. Effect of the Secretome Derived from Transfected UC-MSCs on the Osteogenic Differentiation of Non-Transfected UC-jMSCs

**Figure 6:**
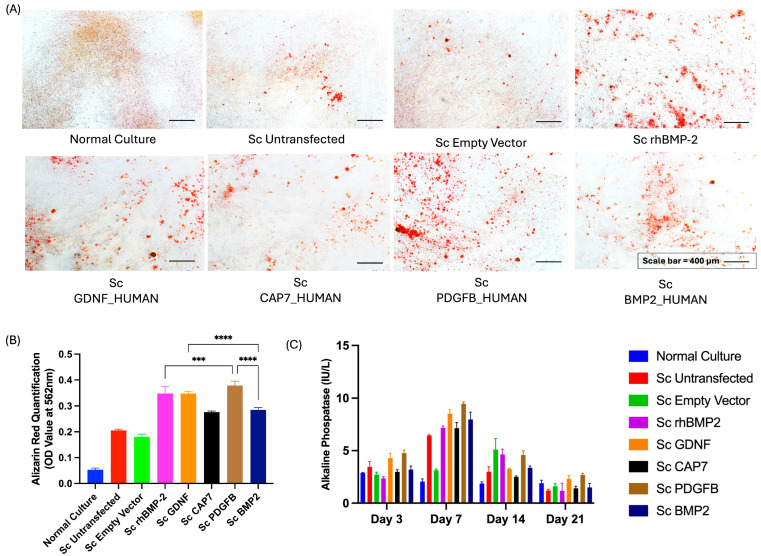
Effect of the secretome derived from transfected UC-MSCs on the osteogenic differentiation of native MSCs: (**A**) Representative images of Alizarin Red staining showing calcium deposition (red) after 21 days of osteogenic differentiation. (**B**) Quantitative analysis of Alizarin Red staining measured at 562 nm. (**C**) ALP activity measured at 3, 7, 14, and 21 days of differentiation. Data are presented as mean ± SD. Statistical significance was determined using one-way ANOVA followed by Tukey’s post hoc test; *p* < 0.001 (***), *p* < 0.0001 (****).

The effects of secretomes derived from transfected UC-MSCs on the osteogenic differentiation of native MSCs were analyzed, and the results are presented in Figure 6. Microscopic observations following Alizarin Red S staining (Figure 6A) revealed the presence of red mineralized nodules, indicative of calcium deposition during osteogenic differentiation. The intensity of these red deposits reflects the extent of calcium accumulation within the extracellular matrix, suggesting the level of osteogenic maturation achieved by each experimental group. Among the groups, cells treated with the secretome from UC-MSCs transfected with PDGFB_HUMAN construct and GDNF_HUMAN construct demonstrated markedly more intense red staining compared to the control and other experimental groups, implying enhanced mineralization activity.

Quantitative analysis of Alizarin Red S staining (Figure 6B) confirmed these qualitative observations. The OD values were significantly higher in the secretome (Sc) PDGFB_HUMAN and Sc GDNF_HUMAN groups compared to the untransfected, empty vector, and normal culture controls (*p* < 0.001–0.0001). These findings indicate that secretomes from transfected UC-MSCs, particularly those expressing osteogenic-related genes, substantially promote matrix mineralization in native MSCs.

The temporal profile of ALP activity (Figure 6C) further supports these observations. ALP levels increased progressively and peaked at day 7 across all groups, consistent with the early phase of osteogenic differentiation. Thereafter, ALP activity declined on days 14 and 21, suggesting the transition from matrix maturation to mineralization. The highest ALP activity was recorded in the Sc GDNF_HUMAN and Sc PDGFB_HUMAN groups, aligning with the enhanced calcium deposition shown by Alizarin Red quantification. Collectively, these results demonstrate that the secretome derived from transfected UC-MSCs exerts a potent inductive effect on osteogenic differentiation in native MSCs.

## 4. Discussion

In this study, UC-MSCs were genetically modified using a conventional plasmid-based transfection method, chosen for its simplicity, low cytotoxicity, and suitability for experiments involving multiple plasmids compared with electroporation or viral approaches [24]. For optimization of transfection efficiency, three different transfection reagents were evaluated: two lipid-based reagents (Lipofectamine 2000^®^ and Lipofectamine 3000^®^) and one polymer-based reagent (PEI). Among these, Lipofectamine 3000^®^ achieved the highest efficiency, approximately 10%, whereas transfection with PEI and Lipofectamine 2000^®^ failed completely, yielding 0% efficiency.

As compared with immortalized cell lines, such as HEK293 and CHO [25], MSCs are generally considered difficult to transfect, with reported transfection efficiencies typically ranging from 0 to 35% [26,27,28,29,30]. This low efficiency is largely attributed to their limited proliferative capacity, which restricts the uptake and intracellular trafficking of DNA–reagent complexes [24,27]. Moreover, the MSC surface is enriched with glycoproteins and proteoglycans that act as physical and electrostatic barriers, limiting the interaction and uptake of transfection complexes [31].

Previous studies have reported similarly low transfection efficiencies in MSCs, with less than 1% using PEI and approximately 2.5% using Lipofectamine 3000^®^ [32]. Several studies have reported that lipid-based transfection reagents provide higher transfection efficiency and better cell viability than polymer-based reagents, both in established cell lines [33,34,35,36] and MSCs [28,32]. Lipid-based transfection utilizes positively charged lipids to bind with negatively charged nucleic acids, forming complexes that can fuse with the cell membrane or be taken up by endocytosis, allowing the genetic material to enter the cell [37].

Cell passage during transfection also determines the success of transfection. In this study, UC-MSCs at passage 2 were used to obtain optimal transfection efficiency, as several studies have reported that higher passage numbers generally correlate with lower transfection efficiency, likely due to changes in cell morphology, behavior, and other characteristics over time [23]. Furthermore, serum-free media such as OPTI-MEM have been shown to enhance transfection efficiency by facilitating the cellular uptake of foreign DNA, particularly when cationic nanoparticles are internalized via endocytosis [38].

Our results showed that different signal peptide constructs lead to different levels of BMP-2 secretion. This finding is consistent with previous studies highlighting the importance of selecting appropriate signal peptides to achieve optimal protein secretion in mammalian cells [12,39,40,41]. SPs such as human azurocidin have been shown to increase the secretion of recombinant interleukin-12 in CHO cells [41]. Other SPs, such as trypsin-1, also increase the secretion of recombinant interleukin-12 in HEK293 cells [42]. Optimal SP selection enhances interaction between nascent proteins with the Signal Recognition Particle (SRP) at the endoplasmic reticulum, leading to more efficient protein maturation and increased extracellular protein release [40].

Interestingly, our study identified PDGFB_HUMAN as SP with the most effective capacity to enhance BMP-2 secretion in UC-MSCs (279.21 ± 6.91 pg/mL). This represents a clear improvement, as a previous study reported BMP-2 concentrations of approximately 0 pg/mL in UC-MSCs’ secretome [43]. In another study using virus-like particles as the delivery vector, the mean BMP-2 concentration in MSCs was reported to be 0.488 pg per cell at 120 h post-transduction [44]. In our study, it was noticed that BMP-2 secretion from UC-MSCs transfected with all plasmid constructs remained transient and gradually decreased with increasing passage number. Transient gene expression is a well-recognized limitation of lipid-based transfection methods. This was also observed in a study in which cells were transfected using a lipid-based reagent, reporting the occurrence of transient expression [45].

After transfection, a reduction in CD105 expression was observed, decreasing from 95–98% to 85–87%, while CD73 and CD90 levels remained >95%. The decrease in CD105 expression in UC-MSCs after transfection may be influenced by several factors. Transfection procedures can induce cellular stress that disrupts membrane integrity and activates stress-related pathways [46]. In addition, high cell confluence after transfection may trigger cell–cell contact inhibition, leading to phenotypic changes [47]. Differentiation of MSCs toward osteogenesis has also been found to decrease CD105 expression [48]. However, decreased CD105 does not automatically impair the function of MSCs. Another study found that CD105-negative MSCs exhibited a much stronger immune modulation capacity related to autocrine production of TGF-β1 by MSCs [49]. CD105-negative MSCs still have multipotency potential, proliferation capacity, and colony-forming capacity similar to the CD105-positive population [50].

Furthermore, our study demonstrated increased secretion of VEGF, TGF-β1, and IL-10 following transfection. This reflects an enhancement of the cells’ angiogenic and immunomodulatory potential, establishing a favorable microenvironment that allows BMP-2 to function optimally [51,52,53]. Activation of BMP-2 signaling may also crosstalk with the canonical Smad 1/5/8 pathway, which functions as a central regulator for multiple processes such as osteogenesis [54], angiogenesis [55], and immunomodulation [56]. Nevertheless, it cannot be excluded that some of these effects may reflect cellular stress responses induced by transfection; further studies, such as assessing markers of oxidative stress, endoplasmic reticulum stress, or apoptosis, are needed to clarify the underlying mechanisms.

To evaluate the osteoinductive potential of the BMP-2 secretome, in vitro osteogenic differentiation was conducted in naive/non-transfected UC-MSCs. The experimental set was adapted from previous research, which used 50% osteogenic induction medium and 50% MSC secretome of total volume [57]. Osteogenic differentiation is a complex process, so it requires other factors that are provided by the osteogenic induction medium. This medium, containing dexamethasone, β-glycerophosphate, and ascorbic acid, provided essential cofactors to promote lineage commitment [58].

Osteogenesis in MSCs proceeds through three main stages. First, during proliferation and osteogenic commitment, cells express transcription factors like RUNX2 and Osterix and show increased ALP activity [59,60]. Next, in the matrix synthesis phase, osteoblasts secrete type I collagen and non-collagenous proteins, such as osteopontin and osteocalcin, forming the extracellular matrix [61]. Finally, mineralization occurs as calcium and phosphate accumulate, producing mineralized nodules detectable by Alizarin Red staining [62]. Our findings demonstrated that the BMP-2 secretome from all genetically modified constructs enhanced osteogenic differentiation significantly more than the native untransfected MSCs’ secretome (Figure 6). This is consistent with the well-established role of BMP-2 as a potent osteoinductive factor that plays a crucial role throughout the stages of osteogenesis by activating Smad signaling pathways, promoting the expression of osteogenic genes, enhancing matrix production, and accelerating mineralization [9].

In this study, the transfection efficiency of the engineered MSCs was transient and only ~10%, which may limit long-term expression and immediate clinical translation. Nevertheless, the results provide important proof-of-concept evidence that optimizing SP sequences can enhance the secretion efficiency of therapeutic proteins such as BMP-2. For clinical scalability, future studies should focus on exploring stable gene delivery strategies, including viral vector-based systems or genome integration approaches, to enable consistent and large-scale production. In addition, safety considerations, such as controlling BMP-2 dosage and minimizing off-target effects, must be carefully evaluated. Finally, in vivo studies are required to validate the therapeutic efficacy, biodistribution, and safety of enhanced BMP-2 secretion before clinical translation.

## 5. Conclusions

This study demonstrated that incorporating specific SPs can significantly enhance BMP-2 gene expression and protein secretion in UC-MSCs. Among the tested SPs, PDGFB_HUMAN exhibited the highest efficiency in promoting extracellular BMP-2 production. Transfection using a lipid-based reagent resulted in a transient protein expression with 10% efficiency. Importantly, the extracellular BMP-2 secreted by engineered UC-MSCs retained its biological activity, effectively promoting osteogenic differentiation of untransfected MSCs based on an in vitro assay. These findings highlight the critical role of SP selection in optimizing recombinant protein secretion from MSCs and offer valuable insights for enhancing the therapeutic potential of stem cell-based strategies in orthopedic and regenerative medicine. Further research should aim to improve transfection efficiency, ensure the long-term stability of engineered MSCs, and explore large-scale production methods to support clinical translation.

## Figures and Tables

**Table 1:** List of signal-peptide sequences.

Signal Peptide	Amino Acid Sequence	Length	Origin Species
GDNF_HUMAN	MKLWDVVAVCLVLLHTASA	19	*Homo sapiens*
CAP7_HUMAN	MTRLTVLALLAGLLASSRA	19	*Homo sapiens*
PDGFB_HUMAN	MNRCWALFLSLCCYLRLVSA	20	*Homo sapiens*
BMP2_HUMAN	MVAGTRCLLALLLPQVLLGGAAG	23	*Homo sapiens*

**Table 2:** Primer sequence for qRT–PCR.

Primer	Sequence (5′ to 3′)
*BMP-2* (NM_001200.4)	F: GAGAAGGAGGAGGCAAAGAAA R: GAAGCTCTGCTGAGGTGATAAA
*GAPDH* (NM_001256799.3)	F: CAATGACCCCTTCATTGACC R: TTGATTTTGGAGGGATCTCG

## Data Availability

The original contributions presented in this study are included in the article/Supplementary Materials. Further inquiries can be directed to the corresponding author.

## Supplementary Materials

The following supporting information can be downloaded at: https://www.mdpi.com/article/10.3390/biomedicines14010076/s1, Table S1: Details of DNA Amounts and Transfection Reagent Volumes Used in Transfection Experiments.

## Abbreviations

ALP	Alkaline Phosphatase
APC	Allophycocyanin
BMP-2	Bone Morphogenetic Protein-2
CAP7	Chemotactic Antibacterial Glycoprotein 7
CHO	Chinese Hamster Ovary
ELISA	Enzyme-Linked Immunosorbent Assay
FITC	Fluorescein Isothiocyanate
FSC-A	Forward Scatter Area
GDNF	Glial-Derived Neurotrophic Factor Human
GFP	Green Fluorescent Protein
HEK293	Human Embryonic Kidney 293
HLA-DR	Human Leukocyte Antigen-DR isotype
IL-10	Interleukin-10
LB	Luria–Bertani
MSCs	Mesenchymal Stem Cells
PDGFB	Platelet-Derived Growth Factor Subunit B
PE	Phycoerythrin
PEI	Polyethyleneimine
qRT-PCR	Quantitative Reverse Transcription Polymerase Chain Reaction
rhBMP-2	Recombinant Human BMP-2
RUNX2	Runt-Related Transcription Factor 2
Sc	Secretome
SP	Signal Peptide
SPFFV	Spleen Focus Forming Virus
SSC-A	Side Scatter Area
SRP	Signal Recognition Particle
TGF-β1	Transforming Growth Factor-beta 1
UC-MSCs	Umbilical Cord-Derived Mesenchymal Stem Cells
VEGF	Vascular Endothelial Growth Factor
