# Supplementary material for: Genetic Engineering of Umbilical Cord-Derived Mesenchymal Stem Cells to Enhance BMP-2 Secretion via Signal Peptide Optimization
Source: Biomedicines. 2025 Dec 30;14(1):76. doi: 10.3390/biomedicines14010076 (PMC12838044; doi:10.3390/biomedicines14010076)
Supplement: Supplementary file 1 [file biomedicines-14-00076-s001.zip › biomedicines-4042254-supplementary.pdf]

Table S1. Details of DNA Amounts and Transfection Reagent Volumes Used in Transfection Experiments

Reagent	Reagent Category	Reagent (µL)/DNA ratio (µg)	Reagent only (µL/well)
PEI	Polymer	3/10	100
Lipofectamine 2000®	Lipid	6/10	100
Lipofectamine 3000®	Lipid	4.5/2.5	100
No Reagent (control)	NA	NA	NA
